# Preliminary ICU experience of a novel intravascular blood glucose sensor

**DOI:** 10.1186/cc10782

**Published:** 2012-03-20

**Authors:** KP Mulavisala, PB Gopal, B Crane, A Mackenzie

**Affiliations:** 1 Axon Anaesthesia Associates Care Hospital Nampally, Hyderabad, India; 2 Apollo Hospitals, Hyderabad, India; 3 Glysure, Abingdon, UK

## Introduction

A need for continuous blood glucose monitoring has always been expressed by critical care practitioners. The results from several iterations of a novel optical fluorescence-based intravascular blood glucose sensor were examined for correlation with an accepted laboratory assay. Ever since Van Den Berghe's group demonstrated reductions in hospital mortality and morbidity from the application of tight glycaemic control [1], many groups have attempted to replicate those results with limited success. Practitioners have speculated upon the reasons behind this observation, and have cited manpower implications and incidence of hypoglycaemic episodes as contributing factors [2]. Investigators have speculated that a continuous blood glucose sensor might contribute towards safe effective glycaemic control [3].

## Methods

A series of postoperative and direct admission ICU patients had an optical fluorescence-based intravascular glucose sensor (GlySure Ltd, Abingdon, UK) placed into the left internal jugular vein on admission to the ICU. The sensor remained *in situ *throughout the ICU stay. Periodic blood samples and simultaneous real-time values of blood glucose measured by the sensor were recorded. The results were correlated with the results of blood sample analysed by a Yellow Springs Instrument glucose analyser. The sensor, which has a heparin coating on its surface, required no further heparinisation; a 'keep vein open' rate of normal saline infusion was maintained throughout the period of operation.

## Results

Sixteen patients received the current configuration blood glucose sensor; during their combined length of stay, 296 paired values were obtained for correlation purposes. A total 99.6% of these values fall within the A+B areas of the Clarke error grid. All sensors continued to function throughout the length of stay, maximum 92 hours, and were withdrawn immediately prior to discharge from the ICU.

## Conclusion

The pre-production intravascular blood glucose sensors successfully track blood glucose values, with improved insight into blood glucose variability in ICU patients.
